# A Longitudinal Analysis of Gambling Predictors among Adolescents

**DOI:** 10.3390/ijerph17249266

**Published:** 2020-12-11

**Authors:** Álvaro Botella-Guijarro, Daniel Lloret-Irles, José Vicente Segura-Heras, Víctor Cabrera-Perona, Juan Antonio Moriano

**Affiliations:** 1 Department of Social and Organizational Psychology, Universidad Nacional de Educación a Distancia (UNED), 28015 Madrid, Spain; alvarbot@cop.es (Á.B.-G.); jamoriano@psi.uned.es (J.A.M.); 2 Health Psychology Department Universidad Miguel Hernández de Elche, 03202 Elche, Spain; vitorperona@gmail.com; 3 Department of Statistics, Mathematics and Informatics Universidad Miguel Hernández de Elche, 03202 Elche, Spain; jvsh@umh.es

**Keywords:** gambling, adolescence, generalized linear model (GLM), risk factor

## Abstract

Although gambling is forbidden for minors, the prevalence of gambling among adolescents is increasing. In order to improve preventive interventions, more evidence on predictors of gambling onset is needed. A longitudinal study was proposed to (1) establish the prevalence of gambling; (2) identify factors associated with gambling behavior the following year; and (3) adjust a model to predict gambling behavior. A cohort of 1074 students (13–18 years old) was followed for 12 months. The prevalence of gambling reached 42.0% in the second measure. Boys gambled 2.7 times more than girls, and the highest percentages of gambling onset showed up between 13 and 14 years old. Gambling onset and maintenance was associated with gender, age, sensation-seeking, risk perception, self-efficacy for not gambling, parents’ attitude towards gambling, group pressure (friends), subjective norm, exposure to advertising, accessibility, normative perception, gambling in T_1_ and parents gambling behavior. Gender, gambling in T_1_ and risk perception were significant in all three logistic adjusted regression models, with the fourth variable being sensation seeking, peer pressure (friends) and accessibility, respectively. It is suggested that universal prevention should be aimed preferably at children under 15 years old and to alert regulators and public administrations to the directly proportional relationship between accessibility and gambling onset.

## 1. Introduction

Gambling is an international phenomenon that involves adolescents and the young population. In Europe, 12–70% of teenagers report having gambled in the last 12 months, with the proportion of excessive gamblers being 15% and problematic gamblers, 5.0% [1,2,3]. In Spain, the prevalence of gambling in the last year among students aged from 14 to 18 varies from 22 to 62% [4,5,6]. The latest ESPAD Report [2] outlines a European average of 22% of adolescents who have gambled in the “last 12 months”. The previous ESPAD survey [7] reports that 14% of European adolescents gambled “at least once in their life”. Although the measure has changed between the two waves, and the interpretation should be taken with caution, the prevalence of gambling has increased among the European population aged 15–16 years. On the other hand, the biennial survey among adolescents carried out by the Spanish Observatory of Drugs and Addictions (OEDA) reports an increase in the prevalence of online gambling from 6.4% in 2016 to 10.3% in 2018 [8]. This high prevalence is due in part to online gambling, which has extended availability and increased capacity to intrude into many online activities and intimate spaces, facilitating a sense of security, immunity and anonymity [9,10]. In Spain, online gambling represents more than 20% of total gambling, with 25.32% of users between 18 and 25 years old [11,12]. In addition, online players have a higher rate of problematic gambling than offline players [13] and bet on more modes of play [14,15]. In adolescence, critical thinking and other cognitive skills are still developing, which makes adolescents more prone to gambling [4,16,17,18,19,20]. In this sense, the prevalence of risk and/or problem gambling among adolescents is higher than in adults [4,17,18,19,20,21].

Early gambling increases the probability of developing addictive behavior as an adult [22,23,24]. Regarding adolescents at-risk gambling, a recent European review [21] shows the highest prevalence rates in Spain, possibly due to recent online gambling legalization and high accessibility to gambling halls. Thus, in Spain, the proportion of nonproblematic gamblers who began gambling before the age of 18 is 13.4% and increases to 44.8% among problematic and pathological gamblers [12]. Since the appearance of online gambling, the proportion of young people who require treatment for gambling addiction has increased, with the main demand being for online gambling [25].

Therefore, carrying out preventive initiatives that target adolescents is of special importance. A conceptual framework for the development of preventive initiatives based on evidence, with broad support in the scientific literature, is Dickson’s model [26,27]. The model suggests that risk and protective factors are specific and common for different risk behaviors. Some of the most common factors are biological (e.g., family history of problem behavior), social environment (e.g., low socioeconomic status), perceived social-environment (e.g., role models for problem behavior), personality (e.g., low self-esteem) and behavior (e.g., other problem behaviors) [28].

The specific risk factors for gambling incorporated into the model are lack of coping skills, cognitive bias and fallacies about gambling, accessibility, permissiveness with underage gambling, and having friends who also gamble [29,30].

St-Pierre and Derevensky [31] classify the programs aimed at adolescents into psychoeducational prevention programs and psychoeducational prevention programs with skills training. The latter include components to reduce gambling misinformation, probability, cognitive bias about gambling, and risk perception. For the skills training, a wide variety of topics are included, such as self-esteem, social and coping skills, decision-making, problem solving and skills to refuse to gamble [16,28,32].

Other authors consider that gambling among adolescents is also predicted by non-reflective processes such as behavior-driven by affordances [33], normative perception and judgment about gambling behavior when it is approved by people who are considered important [34,35] to the individual. Taking the above into account, it is proposed to find the risk factors that predict gambling behavior, among those which make up the components of adolescent gambling prevention programs.

These factors can be classified according to personal, relational, sociodemographic or environmental characteristics. Among the personal features, there is grounded evidence regarding impulsiveness and sensation seeking. Adolescence is a period in which impulse control plays an important role in behavior understanding. Impulsiveness is considered a multidimensional construct, with at least three potentially independent types: Acting without planning or thinking about the consequences, also called lack of premeditation; Showing impatience when deciding between an immediate reward and a bigger but delayed reward; and sensation-seeking being, the tendency to seek new, exciting, or rewarding experiences [36,37]. A wealth of research exists on the relationship between gambling and impulsiveness in cross-sectional [38,39,40,41] and longitudinal studies [22,42,43], as well as the meta-analysis [19]. Other personal risk factors are the low perception of the risks of gambling [43,44], adolescents understand that gambling can carry risks but do not consider themselves as a possible victim [30]; and low self-efficacy for refusing gambling opportunities [43,44,45,46]. Different theoretical models, such as COM-B [47], or the theory of planned behavior [48], assume self-efficacy as a crucial factor for behavior explanation. Regarding family characteristics, there is solid evidence that relates that parent’s attitudes and behavior towards gambling are conducive to adolescents’ gambling behaviors [49,50]. Likewise, parental supervision is a common variable for many risk behaviors [34].

Among the interpersonal or relational factors, two factors stand out: the number of friends who gamble (peer pressure), and the opinion they have about gambling and how important it is for the adolescent (subjective norm). Both factors reinforce the adolescent’s identity in her/his peer group and predispose him/her to imitate the group behaviors [29,30,51], reducing the skills to refuse risk behaviors [52]. Another psychosocial factor is normative perception, understood as the belief that gambling behavior is normal in adolescence [31,53].

From an environmental perspective, exposure to gambling advertising [54,55,56,57] and accessibility to gambling [5,58,59,60,61] are risk factors that increase the probability of gambling in adolescents. Among sociodemographic factors, age and male gender correlate with gambling frequency in the vast majority of studies [4,5,11,21].

Finally, adolescents keep a high fidelity to gambling once they have a first experience [22,43,55,62]. In this sense, the performance of gambling-like behaviors, either with simulated gambling [63,64] or with video games with similar features to online gambling [65], are related to an earlier gambling onset.

Framed into a socioecological model [66], the present study aims to fill the existing gaps in the literature. Although previous longitudinal studies have identified several risk and protective factors, the age ranges are too wide and heterogeneous [67,68], and the goals were to predict problem gambling in adulthood [68,69]. To our knowledge, no study has examined the age range that encompasses the entire adolescence (13–17 years), and no longitudinal studies were conducted in the Spanish population. Since gambling in Spain was regularized in 2011, media pressure has grown enormously, especially in timeframe and channels for children and young people. Unlike previous studies, and in coherence with the components of prevention programs [70,71], this work includes media pressure and advertising among the predictive variables.

Research is needed to identify and delimit factors associated with the problem of gambling, especially those related to social relationships and environmental [19,72]

Cross-sectional studies are the most widely used design method; however, the results of this method only show the association between risk factors and consumption, without the ability to analyze causality. Longitudinal studies are the most powerful design for making causal inferences [73].

The objectives of the present study are (1) to explore the prevalence of gambling from a longitudinal perspective (2) to identify which psychosocial factors predict the initiation and maintenance of gambling behavior in adolescents; and (3) to create a mathematical model to predict gambling behavior. We expect to find prevalences within the range of the mentioned research (Hypothesis 1); the factors studied will all be associated with the initiation or maintenance of gambling behavior in the second year (Hypothesis 2). As for the predictive model, gender and gambling behavior in the first year will be predictive variables of gambling in the second year (Hypothesis 3). Accessibility to gambling and the fact that friends gamble may be other predictors that make up the predictive algorithm (Hypothesis 4).

## 2. Materials and Methods

### 2.1. Participants

The sample was recruited in two consecutive years in 15 high schools in the region of Alicante (Spain). At the time of the first measurement (T_1_), the sample was composed of 2716 students of the third and fourth year of compulsory secondary education (ESO) and 1st year of the baccalaureate (BAC); with an average age of 15.12 (SD = 1.03; range 13–17) and 49.9% girls. At the time of the second measurement (T_2_), 2430 students from the fourth year of ESO, first of the BAC and second of the BAC, participated, with an average age of 16.07 (SD = 0.99; range 14–19), 54.8% girls.

The paired sample T_1_–T_2_ is formed by 1074 students from the 4th year of ESO, and the 1st and second years of BAC, with 55.12% girls and an average age of 15.06 (SD = 1.01; range 13–18). 60.46% of participants from T_1_ and 55.85% of participants from T_2_. The experimental loss was due to (1) the impossibility of measuring students who changed schools or municipalities after completing compulsory education in the next academic year; (2) failure to obtain parental permission and/or absenteeism; (3) errors or falsification in the coding of anonymous keys.

### 2.2. Procedure

After authorization was obtained from the competent authority in education, the study was approved by the Ethics Committee of the Miguel Hernández University (DPS.DLI.01.17). The educational centers were randomly selected, with a ratio of two centers per town, and within each center, all classes from each educational were selected. The valid cases, excluding questionnaires with random pattern or desirability, was up to 85%. Informed consent was obtained from the parents and guardians of the participants. The adolescents participated voluntarily after having been informed of the purpose of the study. No exclusion criteria were used. The duration of the sessions was 25–30 min, and the test was completed collectively, under the supervision of the research team.

### 2.3. Measures

Gambling frequency was informed with a survey adapted from the European school survey project on alcohol and other drugs (ESPAD) questionnaire. The number of times gambled (a) during the lifetime, (b) in the last 12 months, (c) in the last 30 days; in eight gambling modes: online sports betting, sports betting in gambling parlors and/or bars, slot machines in gambling parlors and/or bars, online poker, poker with friends, online casino games, roulette in gambling parlors, and noncommercial betting among peers was measured. Higher scores indicate a higher frequency. The frequency was categorized into four categories: (I) Does not gamble (never played betting games); (II) Occasional frequency (having gambled in online sports betting or slot machines less than five times in a lifetime and less than four times in the last 12 months and never played poker or roulette); (III) Moderate frequency (having gambled at poker or roulette less than three times in a lifetime, sports betting or slot machines less than three times in the last 30 days, less than seven in the last year, and less than eight over a lifetime); and (IV) High frequency (having gambled in the last 30 days more than three times at sports betting or slot machines, or more than twice at online poker or roulette rooms).

For the statistical modeling, we grouped the no gambling and occasional frequency categories into one category (infrequent gambling), while moderate frequency and high frequency were grouped into the frequent gambling category.

Problematic gambling was measured with the SOGS-RA (South Oaks gambling scale-RA) [74,75]. 12 dichotomous items and one Likert-type item. Reliability, Cronbach’s α = 0.72. SOGS-RA scores provide three categories: (1) non-gambler or nonproblematic gambler with scores ≤1; (2) at-risk gambler, with scores between 2 and 3; and (3) problematic gambler, with scores ≥4.

Impulsivity was measured with Plutchik’s impulsivity scale [76,77], consisting of 15 phrases scored on a scale of four alternatives (0 = never, 3 = almost always). Internal consistency: Cronbach’s α = 0.68.

Sensation-seeking was assessed with the brief sensation-seeking scale (BSSS-8) [78,79,80]. An 8-item questionnaire, which evaluates four factors: thrill-seeking and adventure, experience-seeking, disinhibition, and susceptibility to boredom. Internal consistency: Cronbach’s α = 0.76.

Self-efficacy not to gamble (ad-hoc scale) was measured with an 8-item questionnaire that evaluates the ability to not gamble in situations where gambling is encouraged. A 5-option Likert scale was used (0 = non capable, 4 = totally capable). The internal consistency value of Cronbach’s α was 0.85.

Peer pressure was scored with an ad-hoc scale that evaluated the perception of gambling among friends with 8 independent items with the same wording, one for each gamble, in the form, “How many friends do you think that gamble…?”.

Subjective norm was the result of the combination of the perceived opinion about gambling from parents, friends, classmates, teachers, and romantic partners, and the importance that this opinion has for the respondent. We used an ad-hoc questionnaire consisting of 5 items per scale (Likert scale), which were multiplied to obtain the score for this variable (range 0 to 16).

Parents’ attitude towards gambling (8 items), norm perception (4 items), accessibility (6 items) and risk perception (8 items) were measured with the EDGAR-A battery (early detection gambling addiction risk-adolescents) [81]. A 5-point Likert scale was used. Internal consistency: Cronbach α = 0.86 for parental attitude to gambling, 0.80 for norm perception, 0.74 for accessibility, and 0.79 for risk perception.

Parenting gambling behavior was scored using a five-item, ad-hoc questionnaire that assessed whether parents engage in any form of gambling: sports betting, slot machines, poker, casinos, or bingo.

Media pressure was scored with a 12-item Likert scale, which evaluated the perception of exposure to advertising regarding sports betting and casino and online poker from different media outlets such as television, Internet, radio, magazines, outdoor advertising and the presence of gambling parlors in public. The response ranged from 0 (never) to 4 (very often).

### 2.4. Statistical Analysis

A cross-sectional descriptive analysis of variables related to gambling behavior at time points T_1_ and T_2_ (frequency of gambling, intention to gamble, and problem gambling) by gender and age was conducted.

For predictive analysis, continuous variables were represented by their means and standard deviations, while categorical variables were expressed as frequency and percentages. A *t*-test for independent samples was carried out to evaluate the statistical significance of the continuous variables, and a chi-squared test for the categorical variables. For the adjustment of the multivariate logistic regression model, 2/3 (*n* = 721) of the sample (derived or origin sample) was randomly selected. The remaining third (*n* = 353) was used to validate the model (validation sample). This model was used to estimate the probability of “gambling behavior in T_2_”. For its construction, all the variables that presented a *p* < 0.10 value in the univariate analysis were considered. Odds ratios and their 95% confidence intervals were calculated from the coefficients of the final model. To measure the goodness of fit of the model, the Hosmer and Lemeshow (HL) test was performed. Nagelkerke’s R2 value was used to estimate the variability ratio. We tested the assumption of multicollinearity through the variance inflation factor (VIF).

Discrimination and prediction accuracy were assessed by means of the area under the curve (AUC) by calculating the receiver operating characteristic curve (ROC). AUC ranges below 0.5 are considered non-informative and close to 1.0 as perfect prediction models. A *p* < 0.05 value was considered statistically significant. Statistical analysis was performed with R software [82].

We used the forward stepwise regression to select step-by-step, the most important variable. The first three variables, in order of importance, were: Gambling behavior in T_1_, gender and risk perception in T_1_. The R2 Nagelkerke values increased from 0.213 to 0.285 and 0.302, respectively. When we selected the fourth variable: sensation seeking, peer pressure and availability were interchangeable. When the three of them were included, none of them were significant. However, when they were added separately, all three were significant. Therefore, once gender, gambling in T_1_ and risk perception were fixed, we built three logistic regression models. One for each of the other three considered variables. Akaike’s information criterion would recommend that we stick with model C; however, the results are similar in all three; therefore, we considered it relevant to present them all.

## 3. Results

Regarding the frequency of gambling, 28.50% report having played at some time in their lives in T_1_, with one year later (T_2_) it being 42.0%. Table 1 shows the percentages, according to the different frequencies of gambling contemplated, by gender and the total of the sample, for T_1_ and T_2_. The differences in frequency between boys and girls are statistically significant, both in T_1_ (χ^2^ (3, *n* = 2716) = 286.92; *p* < 0.001), and in T_2_ (χ^2^ (3, *n* = 2430) = 400.91; *p* < 0.001). Analyzing the adjusted standardized residuals from the contingency table of the frequency and gender variables, in T_1,_ all the residuals are greater than 1.96 (*p* < 0.05). In T_2_, only those in the frequency categories “do not gamble” and “high” are greater than 1.96 (*p* < 0.05).

**Figure 1:**
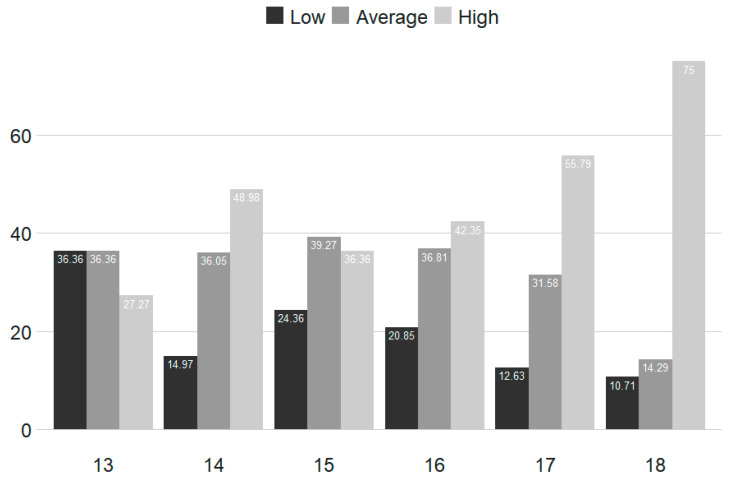
Gambling frequencies by age (%) for T_1_ (*n* = 774 individuals who gambled within the past 12 months).

**Figure 2:**
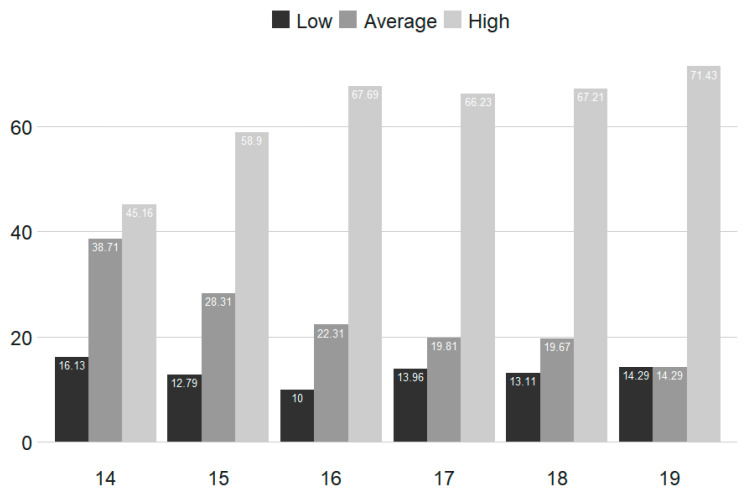
Gambling frequencies by age (%) for T_2_ (*n* = 1023 individuals who gambled within the past 12 months).

Figure 1 and Figure 2 show the graphs for the frequency of gambling among the people who gamble within the past 12 months, in relation to age at T_1_ and T_2_.

**Figure 3:**
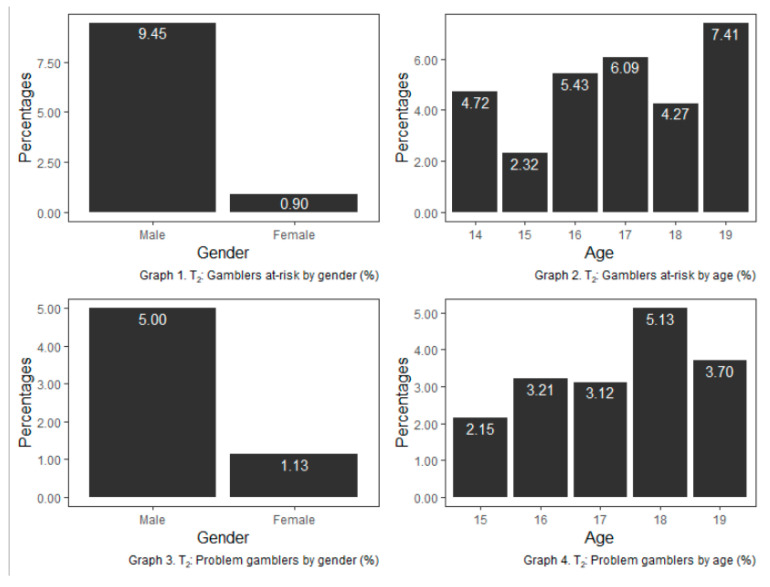
At-risk and problem gamblers by age and gender (%) for T_2_. The percentage refers to both the percentage of total males or females who are classified as a risk (*n* =116) or problem gambler (*n* = 70) and the percentage of total youths of each age who are classified as a risk or problem gambler.

Analysis of the results of problem gambling (SOGS-R) in T_2_ showed 70 cases (2.87%) of problem gamblers and 116 cases (4.76%) for at-risk gamblers. The distribution by gender and age of at-risk and problem gamblers is shown in Figure 3.

On the other hand, with the samples in T_1_ and T_2_ paired, the results show that the highest number of initiations to gamble occurs between 13 and 14 years old.

For the predictive analyses, Table 2 and Table 3 show the results of the univariate analyses of the predictor variables in T_1_ with respect to gambling behavior in T_2_. Except for “impulsivity” and “subjective norm”, all the analyzed factors register higher scores in T_1_ in the adolescents who gamble in comparison to those who do not gamble. The factor “self-efficacy to not gamble” reverses this result since, unlike the others, higher scores indicate a lower risk of gambling.

**Figure 4:**
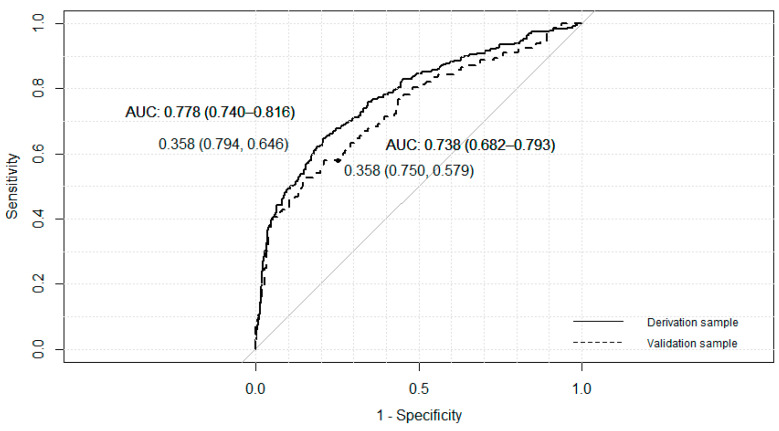
ROC curves for derivative and validation samples, model A, AUC = Area Under the Curve.

**Figure 5:**
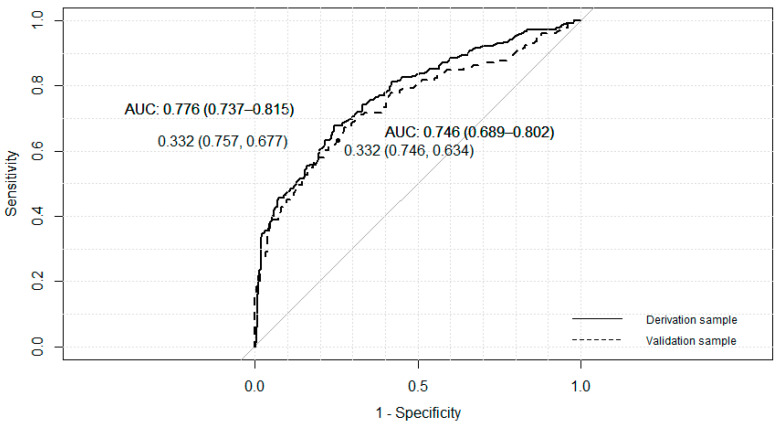
ROC curves for derivative and validation samples, model B, AUC = Area Under the Curve.

**Figure 6:**
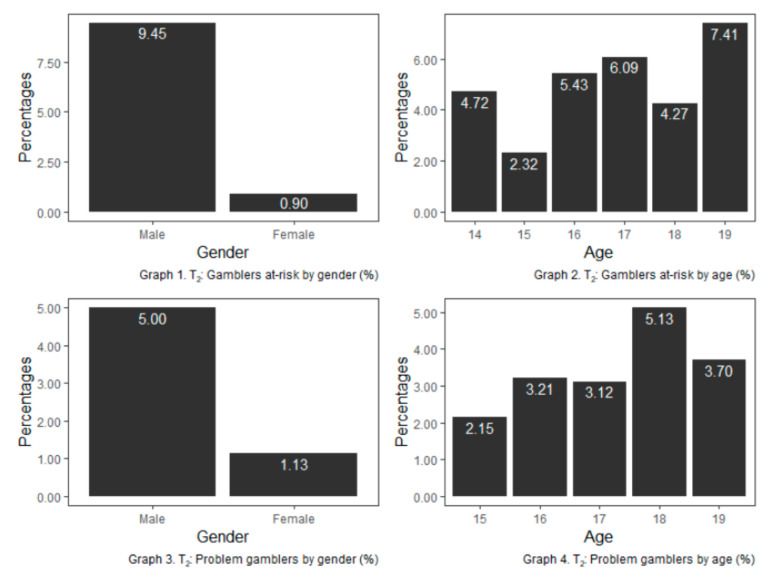
ROC curves for derivative and validation samples, model C, AUC = Area Under the Curve.

The three final models adjusted with the derivation sample identified four significant independent variables that pointed to gambling behavior in T_2_: gambling behavior in T_1_, gender, risk perception, in all models, and sensation seeking, peer pressure or accessibility, depending on the model (Table 4 and Figure 4, Figure 5 and Figure 6). The *p*-value associated with the HL goodness-of-fit test was greater than 0.05, and Nagelkerke’s R2 value was greater than 0.30. Model C showed the lowest Akaike information criterion (AIC).

The final A model, adjusted with the derived sample, shows an area under the ROC curve of 0.778 (95% CI: 0.74–0.82). A cutoff point of 0.358 provided a sensitivity of 64.6% and a specificity of 79.4%. This cutoff point allowed us to rank similar proportions of people in the validation sample, with an area under the curve of 0.738 (95% CI: 0.68–0.79).

The B model, adjusted with the derived sample, shows an area under the ROC curve of 0.776 (95% CI: 0.74–0.82). A cutoff point of 0.332 provided a sensitivity of 67.7% and a specificity of 75.7%. This cutoff point allowed us to rank similar proportions of people in the validation sample, with an area under the curve of 0.746 (95% CI: 0.69–0.80).

Model C, adjusted with the derived sample, shows an area under the ROC curve of 0.775 (95% CI: 0.74–0.82). A cutoff point of 0.325 provided a sensitivity of 69.3% and a specificity of 74.2%. This cutoff point allowed us to rank similar proportions of people in the validation sample, with an area under the curve of 0.744 (95% CI: 0.69–0.80).

The analysis of the possible scenarios for each model is shown in the following figures, which show the scores an individual would have in the model according to his/her circumstances and whether he/she would exceed the threshold (horizontal line) to be classified as positive, i.e., gambling at T_2_. The X-axis collects the values of risk perception (0–4) and the points of the values of the fourth variable in each model.

**Figure 7:**
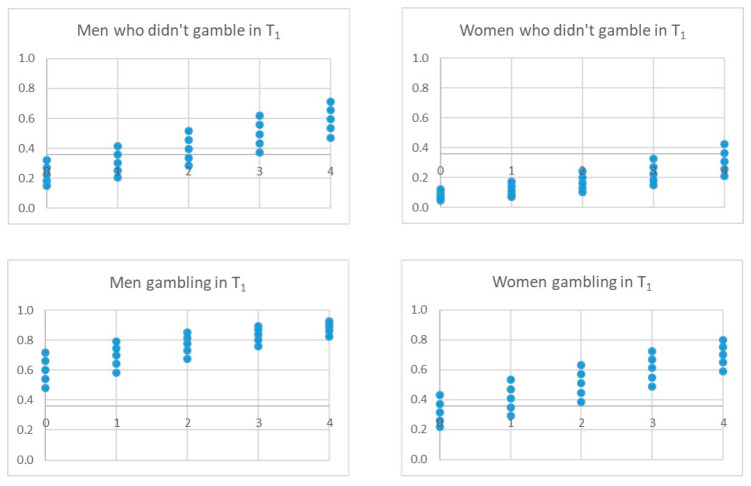
Possible scenarios for model A. Note. The X-axis represents the values of the variable risk perception (0, 4). The dots represent the score on the sensation-seeking variable. The horizontal line represents the threshold for a person to be classified as a gambler or not at T_2_.

Figure 7 shows an analysis of the possible scenarios for model A. Thus, for example, a man who has played in T_1_ would be classified as a gambler in T_2_, regardless of his risk perception and sensation-seeking score; while a woman who has played in T_1_ may be classified as a non-gambler in T_2_ if her risk perception is high and her sensation-seeking score is low.

**Figure 8:**
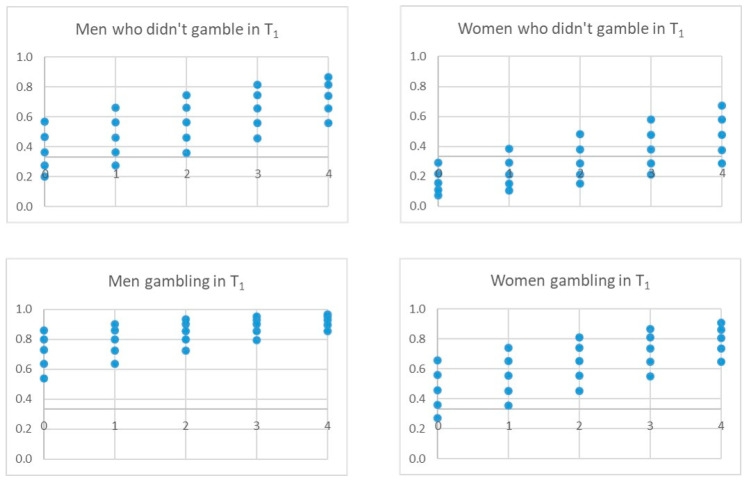
Possible scenarios for model B. Note The X-axis represents the values of the variable risk perception (0, 4). The dots represent the score on the Peer pressure (friends) variable. The horizontal line represents the threshold for a person to be classified as a gambler or not at T_2_.

Figure 8 shows the possible scenarios for model B. For example, the fact that friends gamble can be a determining factor in classifying boys as players in T_2_, without having played in T_1_, even with a perceived high risk. This occurs to a lesser extent in girls.

**Figure 9:**
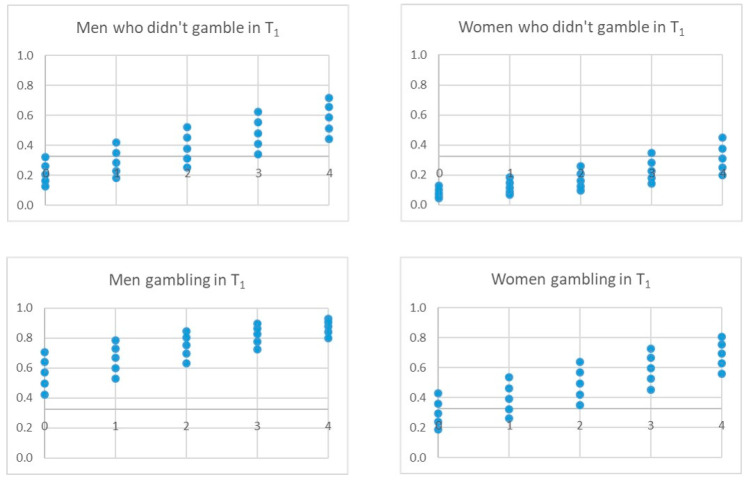
Possible scenarios for model C. Note The X-axis represents the values of the variable risk perception (0, 4). The dots represent the score on the accessibility variable. The horizontal line represents the threshold for a person to be classified as a gambler or not at T_2_.

Figure 9 represents the possible scenarios for model C. For example, the accessibility variable seems to have a greater effect on those who have not gambled in T_1_, both for girls and boys, and little effect on those who have already gambled in T_1_.

## 4. Discussion

The objectives of this study were to determine the prevalence of gambling from a longitudinal perspective, to identify the psychosocial factors that are associated with the onset and maintenance of gambling behavior after one year (T_2_), and to fit a mathematical model (multivariate logistic regression with a longitudinal approach) to predict gambling behavior from these psychosocial factors.

This work supposes a rational approach to the greater knowledge of the predictive factors that can act in the onset and maintenance of gambling in adolescents. Thus, it represents an advance in our country, as it is a pioneering study that combines from a longitudinal perspective different levels of individual and psychosocial risk factors, and includes factors typically considered as components of school-based preventive programs (i.e., risk perception, peer pressure, media pressure, etc.) [70,71].

Regarding the first objective, the gambling frequency rates at T_1_ are similar to those reported by the Survey ESTUDES 2018 [8], both for the total results and for the trends according to age and gender. At T_2_, the average age of the sample increases by one year, and the percentage of young people who gamble with high frequency practically doubles. Between 13 and 14 years old, 45% of young people who did not gamble in T_1_ start gambling. These results are consistent with those found in similar studies [83,84] and invite to reflect on the appropriate age to carry out preventive interventions.

Our results show a stable increase in gambling in adolescents aged 13–17 years. These findings contrast with other longitudinal studies such as those carried out by Delfabbro, King and Griffiths [67], which indicate that gambling shows little stability in young people, perhaps because their age ranges are between 16 and 21 years, encompassing the transition to young adulthood. Other studies suggest that there is an increasing trend in gambling, the prevalence growing rapidly in adolescents to decline in young adulthood [69,85].

Gambling presents a sexual dimorphism in high frequencies of gambling, where the male-female ratio is 5:1. This difference is reduced at T_2_ when the average age is one-year-older, suggesting that girls who gamble at high frequencies do it later than boys. For low and moderate frequencies, the gender differences decrease, becoming statistically no-significant. The rates of risk gambling (4.76%) or problem gambling (2.87%) and the difference between boys and girls are in line with those found in other similar studies [2,4,17] and are higher than those found in studies of the adult population [12].

The univariate analyses of the predictive modeling of gambling behavior at T_2_ from the scores in the predictor variables at T_1_ exclude the variables “impulsivity” and “subjective norm” since they do not show statistically significant differences between the group “Does not gamble” and “Occasional Frequency” at T_2_. These results contradict the second hypothesis that predicts that all proposed factors will be associated with the initiation or maintenance of gambling behavior in the second year.

The analysis of the risk factors casts three models with similar predictive capacity. The models do not include interactions between variables to avoid complexity and difficulty of interpretation. The three models share three of the four predictive variables in T_1_: gender, gambling behavior at T_1_ and risk perception. Hypothesis 3, which predicted gender and gambling behavior in the first year as predictive variables for gambling in the second year, is verified. As mentioned above, studies of gambling find a strong sexual dimorphism, so it was expected that being male would be one of the predictive variables of the models. Previous studies have found high rates of continuity with respect to the previous behavior, so it is plausible to think that gambling behavior in the previous year is a good predictor [19,86]. The third common variable is “Risk perception”. The systematic review by Spurrier and Blaszczynski [60] indicates that the most problematic gamblers perceived the game to be less harmful than non-gamblers or problem gamblers. Therefore, increasing risk perception should be a goal of preventive interventions. Taking into account that risk perception becomes more resistant when the person has experienced the behavior, according to the results of our study, it would be desirable to carry out prevention programs between the ages of 13 or 14 years, before adolescents have their first gambling experiences.

In addition to the variables shared by the three models, model A included the variable “sensation-seeking”, model B included “peer pressure,” and model C “accessibility”. All three are variables that appear as risk factors for adolescent gambling in various studies [17,38,55,87,88,89]. The commonality of the three models is shaped by a behavior variable: “frequency of gambling,” a demographic variable: “gender”, and a cognitive variable: “risk perception”. The variable “sensation-seeking” characterizes the A model as a predictive model from the individual perspective. This result is in line with what was observed in recent longitudinal studies and systematic reviews [19,90]. The variable “peer pressure” characterizes model B as a predictive model from the immediate environment. Consistent with this result, other studies have previously shown that boys who gamble at a high-frequency often report the highest level of peer pressure [41]. Moreover, the variable “accessibility” characterizes model C as an environmental predictive model, named as “availability” in previous works; it has been identified as a risk factor, especially for online gambling [21].

The longitudinal design and sample size offer robustness to the study; however, a 12 months period between measures is a short period to examine the evolution of gambling habits over time. In general, the findings should be interpreted under the limitations of self-reporting and cohort studies, including possible biases and attrition of the participants between measures. In this sense, the loss of 40% of the sample in the follow-up invites to reflect on its impact on the results and its validity. Likewise, it would have been desirable to include minors not included in the regular educational system. Although they are a minority, their unique sociodemographic features would enrich the results. Finally, the disadvantages of the concentrated location of the sample must be taken into account and require caution in generalizing results.

The results of this study contribute to a better knowledge of the factors that predict the onset and maintenance of gambling in the adolescent population, reinforce the multidimensional definition of gambling behavior and the need to take into account individual, psychosocial and environmental factors in the design of preventive interventions [91,92,93].

The continuity rate is worrying, with 70% of adolescents who gamble continuing to do so a year later. These findings suggest that universal prevention programs should target children under the age of 15 to anticipate the age of onset. Regarding the factors commonly proposed as components of preventive interventions (i.e., risk perception, attitude change, the illusion of control, media pressure) [70,71], our study supports that prevention programs should target risk perception and resistance to peer pressure. In this sense, other studies in our country confirm low levels of risk perception and that the usual motivation among adolescents in the initial stages of gambling is to feel part of the peer group [94]. Moreover, the identification of individual variables such as sensation-seeking and the ability to resist peer pressure is a clear caveat for designing prevention programs. In addition, the perception of high accessibility must be understood as an alert for the need for community and environmental preventive approaches, with the participation of public administrations and civil society, in compliance with the rules and regulation of media pressure. In this sense, the results indicate the need to implement initiatives that favor compliance with current legislative measures. Gambling venue and website liability could be increased through the implementation of specific normative strategies and actions. Some of these measures are to implement more effective access control systems, also online. These rules and actions may apply to the establishment owner or staff. These types of actions have been effective in preventing alcohol abuse [95].

## 5. Conclusions

In conclusion, our study highlights the role of risk perception, sensation-seeking, peer pressure and accessibility as psychosocial variables predicting the onset and maintenance of play among adolescents. These variables should be taken into account as objectives in the design of preventive interventions. In terms of age of onset and high continuation rate, we suggest that universal prevention programmes should target children under 15 years of age to anticipate the age of onset.

## Figures and Tables

**Table 1:** Gambling frequencies by gender and totals for T_1_ & T_2._

	T_1_ (*n* = 2716)			T_2_ (*n* = 2430)		
	Male	Female	Total	Male	Female	Total
Never	53.6	81.4	71.5	39.27	73.3	57.9
Low	7.5	5.5	10.7	4.27	5.9	5.2
Average	14.8	8.6	4.5	9.82	9.6	9.7
High	24.1	4.5	13.3	46.6	11.2	27.2

**Table 2:** Average scores for infrequent gamblers and gamblers and *p*-value for the t-test of differences between groups.

	Gambling Behavior for T_2_				95% CI	*p*-Value
	Infrequent Gambling		Frequent Gambling			
	*n*	Mean ± SD	*n*	Mean ± SD		
Age	707	14.99 ± 1.00	364	15.18 ± 1.02	(−0.32, −0.06)	0.003
Sensation-seeking	708	3.29 ± 0.77	366	3.42 ± 0.75	(−0.23, −0.04)	0.007
impulsivity	708	2.35 ± 0.36	366	2.39 ± 0.38	(−0.08, 0.01)	0.124
Risk perception	696	1.44 ± 0.64	362	1.73 ± 0.75	(−0.38, −0.20)	<0.001
Self-efficacy not to gamble	693	3.49 ± 0.66	354	3.29 ± 0.79	(0.11, 0.30)	<0.001
Parents attitude	677	0.87 ± 0.71	348	1.05 ± 0.87	(−0.29, −0.08)	0.001
Group pressure (friends)	678	0.44 ± 0.52	352	0.75 ± 0.68	(−0.39, −0.23)	<0.001
Subjective norm: parents	702	4.83 ± 2.82	359	5.61 ± 3.46	(−1.19, −0.36)	<0.001
Subjective norm: friends	701	7.20 ± 3.57	358	7.89 ± 4.16	(−1.20, −0.19)	0.007
Subjective norm: peers	699	5.17 ± 3.10	358	5.25 ± 3.42	(−0.50, 0.35)	0.721
Subjective norm: teachers	700	4.03 ± 2.60	359	3.68 ± 2.74	(0.01, 0.69)	0.041
Subjective norm: partner	686	6.04 ± 3.45	355	6.95 ± 4.00	(−1.4, −0.42)	<0.001
Exposure to publicity	706	24.18 ± 6.36	366	26.3 ± 6.72	(−2.94, −1.30)	<0.001
Accessibility	659	2.41 ± 0.68	345	2.63 ± 0.66	(−0.31, −0.13)	<0.001
Normative perception	696	2.00 ± 0.75	353	2.31 ± 0.88	(−0.41, −0.20)	<0.001

SD = standard deviation. CI = Confidence Interval

**Table 3:** Distribution for gambling behavior at T_2_ compared to T_1_ and *p*-value according to chi-squared.

	Gambling Behavior at T_2_		*p*-Value
	Infrequent Gambling *n* (%)	Frequent Gambling *n* (%)	
Gambling behavior at T_1_			<0.001
No gamble	649(76.4)	201(23.6)	
Yes gambles	59(26.3)	165(73.7)	
Gender			<0.001
Male	234(48.5)	248(51.5)	
Female	474(80.1)	118(19.4)	
Gambling behavior of parents			0.002
Do not gamble	574(68.3)	266(31.7)	
At least one gambles	134(57.3)	100(42.7)	

**Table 4:** Adjusted multivariate logistic regression models.

Variable	Beta	*p*-Value	OR	95% CI	VIF	AIC	R2 Nagelkerke	HL
Model A						725.36	0.308	0.933
Constant	−2.013							
Gambling behavior at T_1_	1.680	<0.001	5.364	3.48–8.34	1.03			
Gender	−1.207	<0.001	0.299	0.21–0.43	1.04			
Risk perception	0.412	0.003	1.509	1.15–1.99	1.02			
Sensation-seeking	0.252	0.046	1.287	1.01–1.65	1.03			
Model B						693.14	0.302	0.776
Constant	−1.374							
Gambling behavior at T_1_	1.525	<0.001	4.596	2.92–7.31	1.10			
Gender	−1.154	<0.001	0.315	0.22–0.46	1.02			
Risk perception	0.401	0.004	1.493	1.14–1.97	1.01			
Peer pressure (friends)	0.412	0.018	1.510	1.07–2.13	1.08			
Model C						684.45	0.302	0.669
Constant	−1.927							
Gambling behavior at T_1_	1.615	<0.001	5.027	3.23–7.90	1.04			
Gender	−1.150	<0.001	0.317	0.22–0.46	1.02			
Risk perception	0.422	0.004	1.524	1.15–2.03	1.02			
Accessibility	0.294	0.040	1.341	1.02–1.78	1.02			

OR: odds ratio; CI = confidence interval; VIF: variance inflation factor. HL = Hosmer and Lemeshow test. AIC: Akaike Information Criterion.
